# Ethanol Metabolism Modifies Hepatic Protein Acylation in Mice

**DOI:** 10.1371/journal.pone.0075868

**Published:** 2013-09-20

**Authors:** Kristofer S. Fritz, Michelle F. Green, Dennis R. Petersen, Matthew D. Hirschey

**Affiliations:** 1 Department of Pharmaceutical Sciences, University of Colorado Denver Anschutz Medical Campus, Aurora, Colorado, United States of America; 2 Sarah W. Stedman Nutrition and Metabolism Center, Duke University Medical Center, Durham, North Carolina, United States of America; 3 Department of Medicine and Department of Pharmacology & Cancer Biology, Duke University Medical Center, Durham, North Carolina, United States of America; Nihon University School of Medicine, Japan

## Abstract

Mitochondrial protein acetylation increases in response to chronic ethanol ingestion in mice, and is thought to reduce mitochondrial function and contribute to the pathogenesis of alcoholic liver disease. The mitochondrial deacetylase SIRT3 regulates the acetylation status of several mitochondrial proteins, including those involved in ethanol metabolism. The newly discovered desuccinylase activity of the mitochondrial sirtuin SIRT5 suggests that protein succinylation could be an important post-translational modification regulating mitochondrial metabolism. To assess the possible role of protein succinylation in ethanol metabolism, we surveyed hepatic sub-cellular protein fractions from mice fed a control or ethanol-supplemented diet for succinyl-lysine, as well as acetyl-, propionyl-, and butyryl-lysine post-translational modifications. We found mitochondrial protein propionylation increases, similar to mitochondrial protein acetylation. In contrast, mitochondrial protein succinylation is reduced. These mitochondrial protein modifications appear to be primarily driven by ethanol metabolism, and not by changes in mitochondrial sirtuin levels. Similar trends in acyl modifications were observed in the nucleus. However, comparatively fewer acyl modifications were observed in the cytoplasmic or the microsomal compartments, and were generally unchanged by ethanol metabolism. Using a mass spectrometry proteomics approach, we identified several candidate acetylated, propionylated, and succinylated proteins, which were enriched using antibodies against each modification. Additionally, we identified several acetyl and propionyl lysine residues on the same sites for a number of proteins and supports the idea of the overlapping nature of lysine-specific acylation. Thus, we show that novel post-translational modifications are present in hepatic mitochondrial, nuclear, cytoplasmic, and microsomal compartments and ethanol ingestion, and its associated metabolism, induce specific changes in these acyl modifications. These data suggest that protein acylation, beyond protein acetylation, contributes to the overall metabolic regulatory network and could play an important role in the pathogenesis of alcoholic liver disease.

## Introduction

Ethanol is metabolized by alcohol dehydrogenases (ADH) to form acetaldehyde, and then by aldehyde dehydrogenases to form acetate with subsequent formation of acetyl-CoA. Acetaldehyde is highly reactive, toxic and immunogenic, and high levels from excessive ethanol consumption can damage cellular proteins and DNA [1-5]. Furthermore, ethanol metabolism reduces the NAD^+^/NADH ratio within the cytosol and mitochondria and could contribute to the reduction in enzymatic activity of NAD^+^-dependent enzymes [6-8].

The sirtuins are a family of NAD^+^-dependent protein deacylases [9], which regulate metabolism and cell survival [10]. Mammals have seven sirtuins (SIRT1-7), which vary in their sub-cellular localization, activity and specificity. Because the sirtuins remove acetyl groups from proteins, loss of the sirtuin activity can lead to protein hyperacetylation. Protein acetylation is a highly abundant post-translational modification [11], which is found on virtually every metabolic protein [12,13]. Hyperacetylation of proteins has been shown to reduce enzymatic activity, specifically of metabolic proteins involved in fatty acid oxidation [14], ketone body synthesis [15], and the urea cycle [16].

In the mitochondria, SIRT3 is the major regulator of protein acetylation levels [17]. *Sirt3* ^*-/-*^ (SIRT3KO) mice show marked mitochondrial protein hyperacetylation, whereas mice lacking *Sirt4* or *Sirt5* show no increases in protein acetylation [17]. Under basal conditions, SIRT3KO mice are metabolically normal [17], but under stressed conditions, show reduced fatty acid oxidation and develop metabolic abnormalities, such as hepatic steatosis, reduced ATP levels, and intolerance to cold exposure [14]. Long-chain acyl-CoA dehydrogenase (LCAD) is an enzyme in the fatty acid oxidation pathway, and is hyperacetylated and displays reduced activity in SIRT3KO mice. Additionally, SIRT3KO mice fed a high-fat diet develop a severe metabolic phenotype, including diet-induced obesity, hyperlipidemia, insulin resistance, hyperinsulinemia, non-alcoholic steatohepatitis, and the metabolic syndrome [18].

Recent studies have reported mitochondrial protein acetylation increases in response to ethanol ingestion in mice [19], and implicates protein post-translational modifications in the regulation of ethanol metabolism. Indeed several of the proteins involved in ethanol metabolism are acetylated, including those involved in lipid metabolism, antioxidant response, amino acid metabolism and energy production by oxidative phosphorylation [20]. Furthermore, in the absence of SIRT3, mice fed an ethanol-containing diet have elevated mitochondrial protein acetylation levels compared to wild-type mice, suggesting SIRT3 could mediate the acetylation status of some of the hyperacetylated proteins [20].

SIRT5 is another mitochondrial sirtuin and in contrast to its previously reported weak deacetylase activity [21], was recently reported to be a strong desuccinylase and demalonylase [22]. While the biological significance of these modifications is not yet known, SIRT5 is known to regulate activity of the urea cycle [23]. Because acetylation has an important role in ethanol metabolism, we hypothesized that protein succinylation could be an important post-translational modification regulating mitochondrial metabolism [24]. To begin to understand this new post-translational modification, we assessed the possible role of protein succinylation in ethanol metabolism by surveying the post-translational modification status from livers of mice fed a control or ethanol-supplemented diet. In addition to succinyl-lysine, we surveyed acetyl-, propionyl-, and butyryl-lysine – other recently described post-translational modifications [24,25] – to gain insight into the global post-translational modification landscape and the changes induced by ethanol metabolism.

## Results

### Mitochondrial acylation during chronic ethanol ingestion

**Figure 1:**
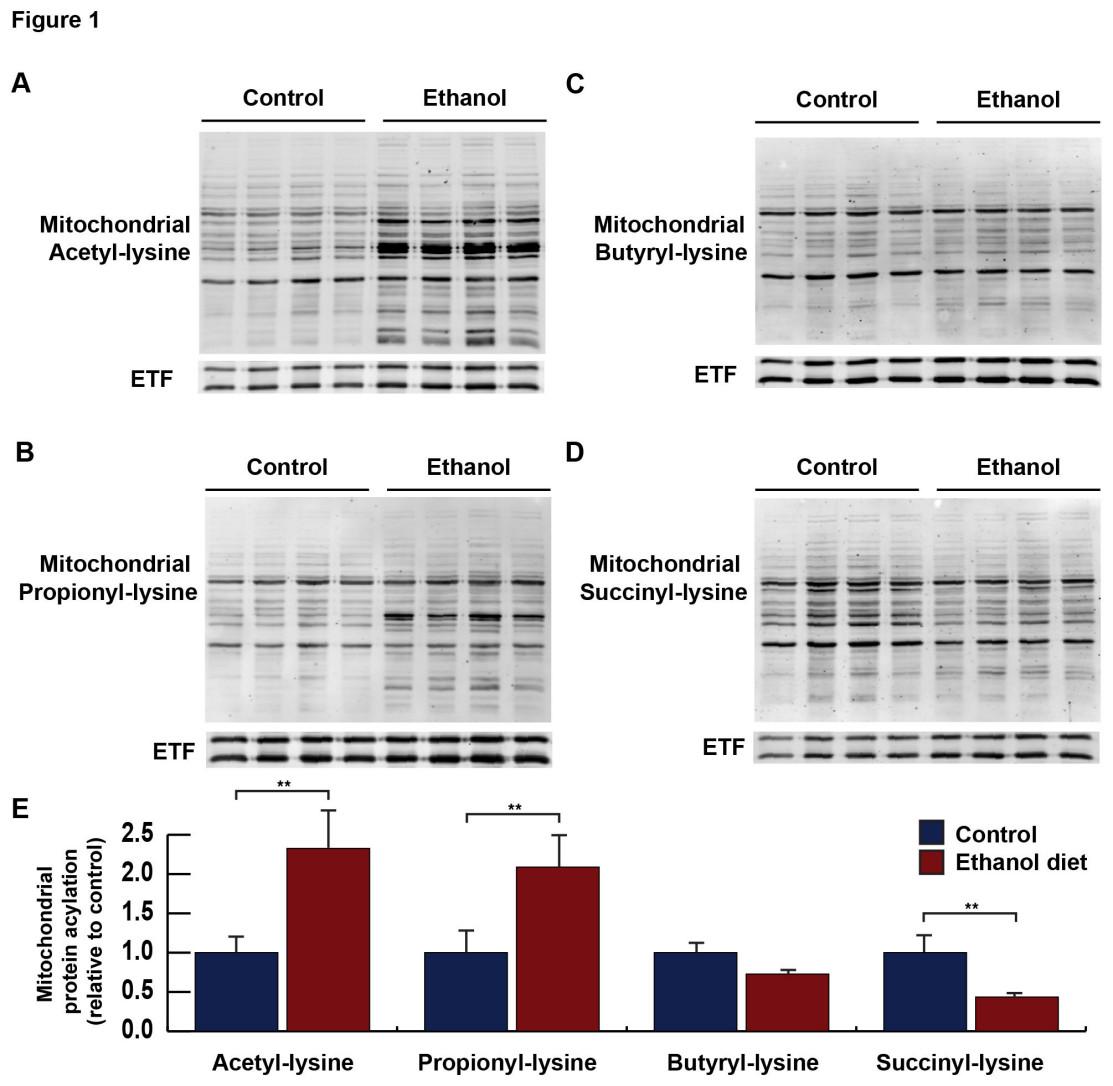
Mitochondria were isolated from livers of wild-type mice fed a standard or ethanol diet for 6-8 weeks and analyzed for mitochondrial protein acetylation (A), propionylation (B), butyrylation (C), and succinylation (D) by western blot analysis with an acyllysine-specific antibody; normalized to total mitochondrial content using anti-electron transfer flavoprotein (ETF); n = 4 mice/condition.

Immunoblot analysis with an anti-acetyllysine antibody of hepatic mitochondrial extracts from mice fed an ethanol-diet or control diet revealed that the ethanol diet induced an approximately 2- fold increase in protein acetylation (Figure 1A and 1E), as previously reported [19]. The same immunoblot analysis with a propionyl-lysine antibody revealed mitochondrial protein propionylation also increases (approximately 2-fold) during ethanol-diet feeding (Figure 1B). In contrast, no changes were observed in mitochondrial protein butyrylation, and the overall butyrylation signal was low, suggesting this modification was not abundant in the mitochondria enough to be detected by this antibody (Figure 1C). When measuring mitochondrial protein succinylation, we observed a marked reduction in succinyl-lysine antibody signal in ethanol-fed mice compared to standard diet control (Figure 1D).

**Figure 2:**
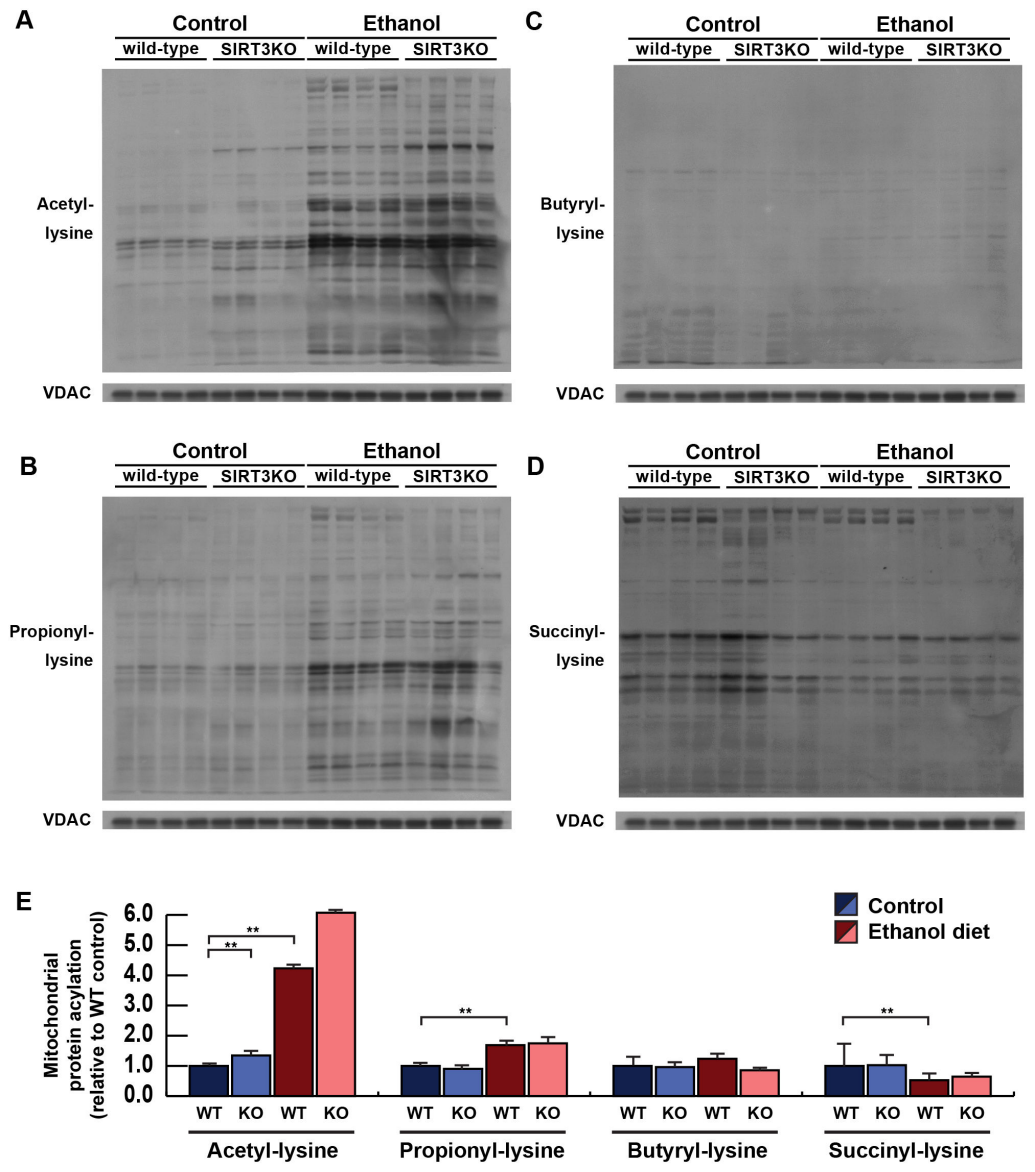
Mitochondrial extracts were isolated from livers of wild-type or SIRT3KO mice fed a standard or ethanol diet for 6-8 weeks and analyzed for total protein acetylation (A), propionylation (B), butyrylation (C), and succinylation (D) by western blot analysis with an acyllysine-specific antibody; normalized to total mitochondrial content using anti-voltage-dependent anion channel (VDAC); n = 4 mice/condition.

SIRT3 mediates the deacetylation of several mitochondrial proteins under standard diet conditions [14,17,26] and ethanol diet conditions [20]. Furthermore, SIRT3 has been shown to depropionylate proteins *in vitro* [27]. To test a role for SIRT3 in mediating the removal of acetylation, propionylation, butyrylation, and succinylation modifications during standard diet or ethanol diet feeding, we measured protein acylation in mitochondrial protein lysates collected from wild-type mice or mice lacking SIRT3 (SIRT3KO) fed a standard or ethanol-containing diet. Hyperacetylation of mitochondrial proteins in SIRT3KO mice is well established [17], and we observed further increases in response to ethanol metabolism, suggesting a role for SIRT3 in ethanol metabolism (Figure 2A), as previously described [20]. To determine the possible role for SIRT3 in regulating post-translational modifications in addition to acetylation, we measured propionylated lysine residues by immunoblotting. We found that while the signal was not strong in wild-type mice under standard conditions, some bands showed elevated signals in the SIRT3KO (Figure 2B), suggesting SIRT3 might regulate this modification *in vivo*. When SIRT3KO mice were fed an ethanol diet, lysine propionylation increased (Figure 2B), as described above (Figure 2B), and this modification was moderately elevated in mice lacking SIRT3 (Figure 2B). In contrast to acetylation and propionylation, lysine butyrylation was unchanged in the liver mitochondria of wild-type and SIRT3KO mice (Figure 2C), and this signal showed no marked changes upon ethanol feeding. These data suggest SIRT3 does not regulate butyrylation of lysine residues in the mitochondria. Additionally, no changes in succinylation were observed in the mitochondria of wild-type compared to SIRT3KO mice under standard diet or ethanol diet feeding, suggesting SIRT3 does not regulate this modification (Figure 2D), as described previously [22].

**Figure 3:**
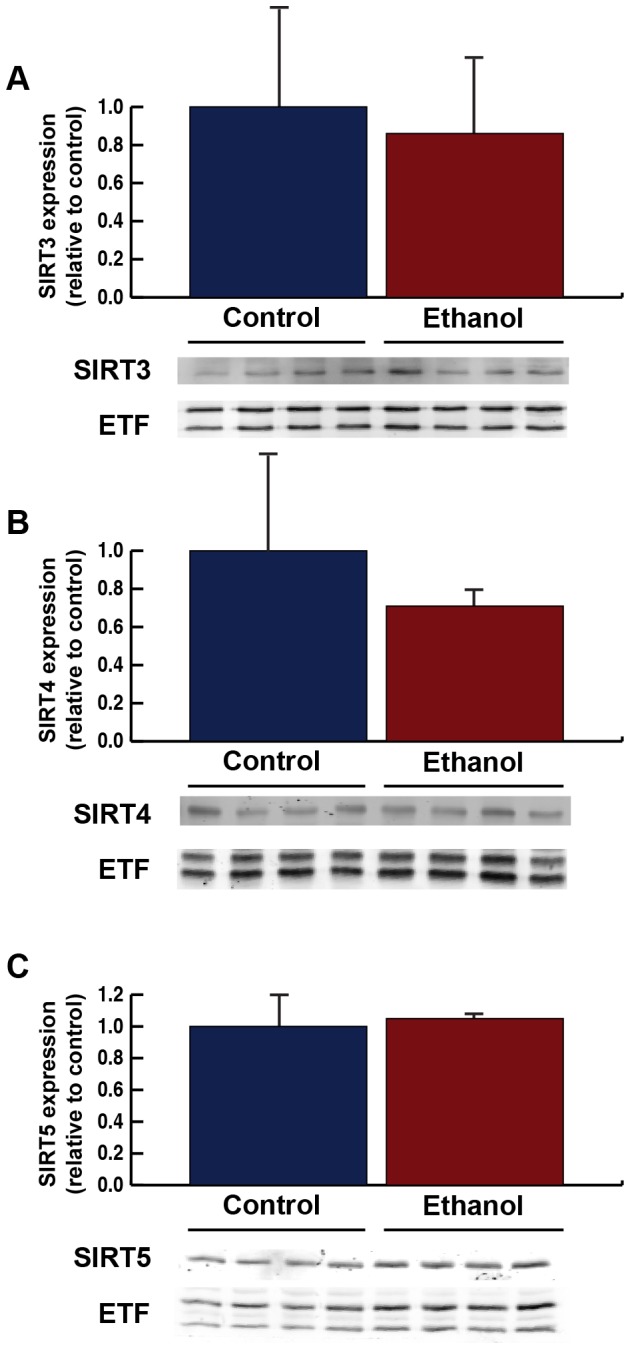
Mitochondria were isolated from livers of wild-type mice fed a standard or ethanol diet for 6-8 weeks and analyzed for mitochondrial SIRT3 (A), SIRT4 (B) and SIRT5 (C) expression; normalized to total mitochondrial content using anti-electron transfer flavoprotein (ETF); n = 4 mice/condition.

To test if alterations in the levels of the mitochondrial sirtuins could be mediating the changes in protein acylation, we measured total mitochondrial SIRT3, SIRT4 and SIRT5. As previously described [19], we observed no changes in SIRT3 protein expression in response to ethanol ingestion (Figure 3A). Additionally, no changes in SIRT4 were observed (Figure 3B). When measuring mitochondrial SIRT5, we found chronic ethanol consumption did not change hepatic SIRT5 (Figure 3C), in contrast to previous reports of its reduction in rats [28]. Together, these data suggest the changes in protein acylation were not due to changes in mitochondrial sirtuin expression.

**Figure 4:**
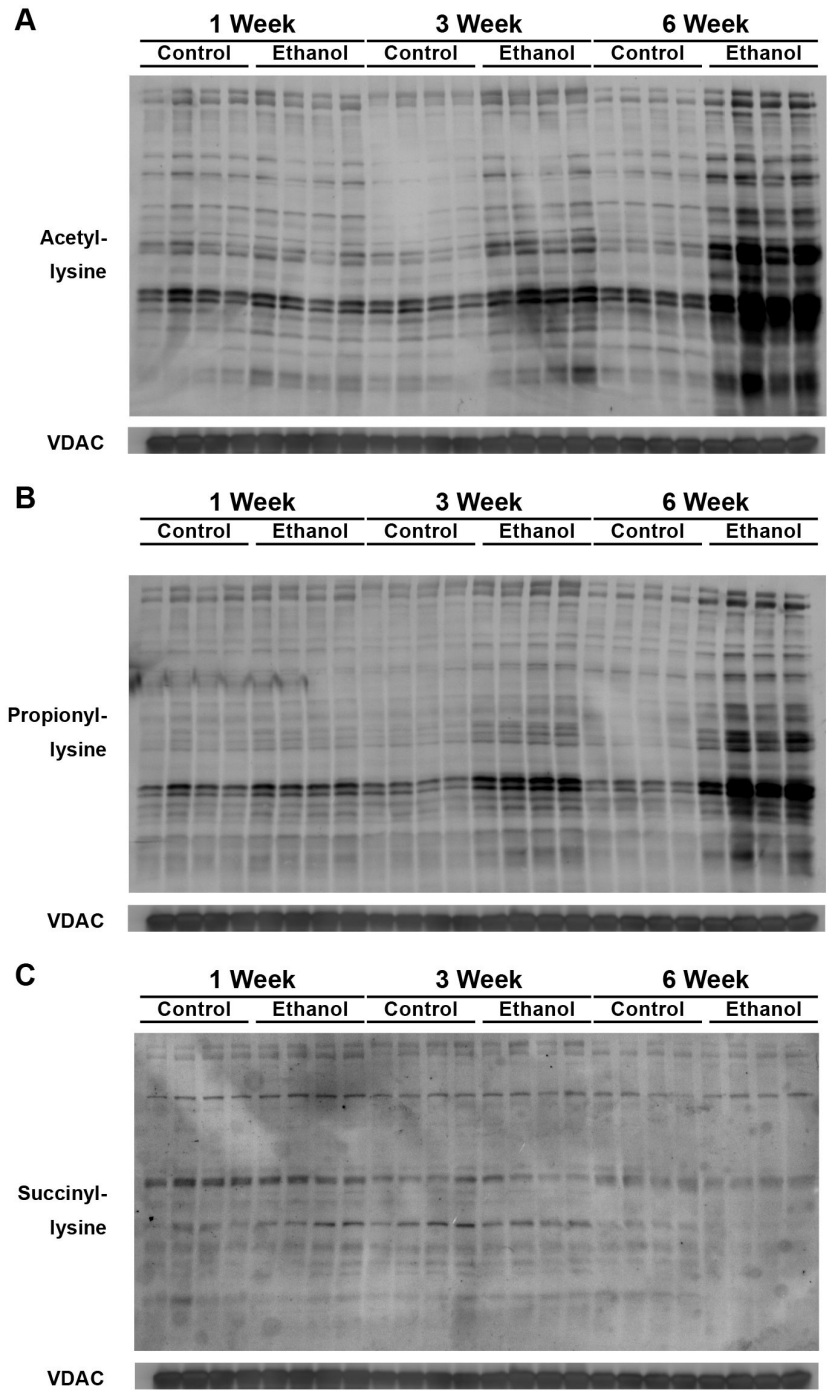
Mitochondria were collected from livers of wild-type mice fed a standard or ethanol diet for 1, 3, or 6 weeks and analyzed for total protein acetylation (A), propionylation (B), and succinylation (C) by western blot analysis with acyllysine-specific antibodies; normalized to total mitochondrial content using anti-voltage-dependent anion channel (VDAC); n = 4 mice/condition.

To further define the ethanol-induced changes in mitochondrial protein acylation, we measured the change in these modifications over time. Wild-type mice were fed an ethanol diet or control diet for 1 week, 3 weeks, or 6 weeks, and were measured for changes in hepatic mitochondrial protein acylation by western blotting (Figure 4). We observed an increase in mitochondrial protein acetylation with prolonged time on the ethanol diet (Figure 4A), consistent with previous reports [6]. Furthermore, we observed a time-dependent increase in mitochondrial protein propionylation (Figure 4B), which paralleled the increase in mitochondrial protein acetylation. When measuring mitochondrial protein succinylation, we observed a time-dependent decrease in this modification (Figure 4C). Together, these data show the changes induced in mitochondrial protein acylation by ethanol metabolism are time-dependent, and suggest that chronic ethanol exposure alters the mitochondrial protein acylome in a dose-dependent manner.

### Nuclear acylation during ethanol metabolism

**Figure 5:**
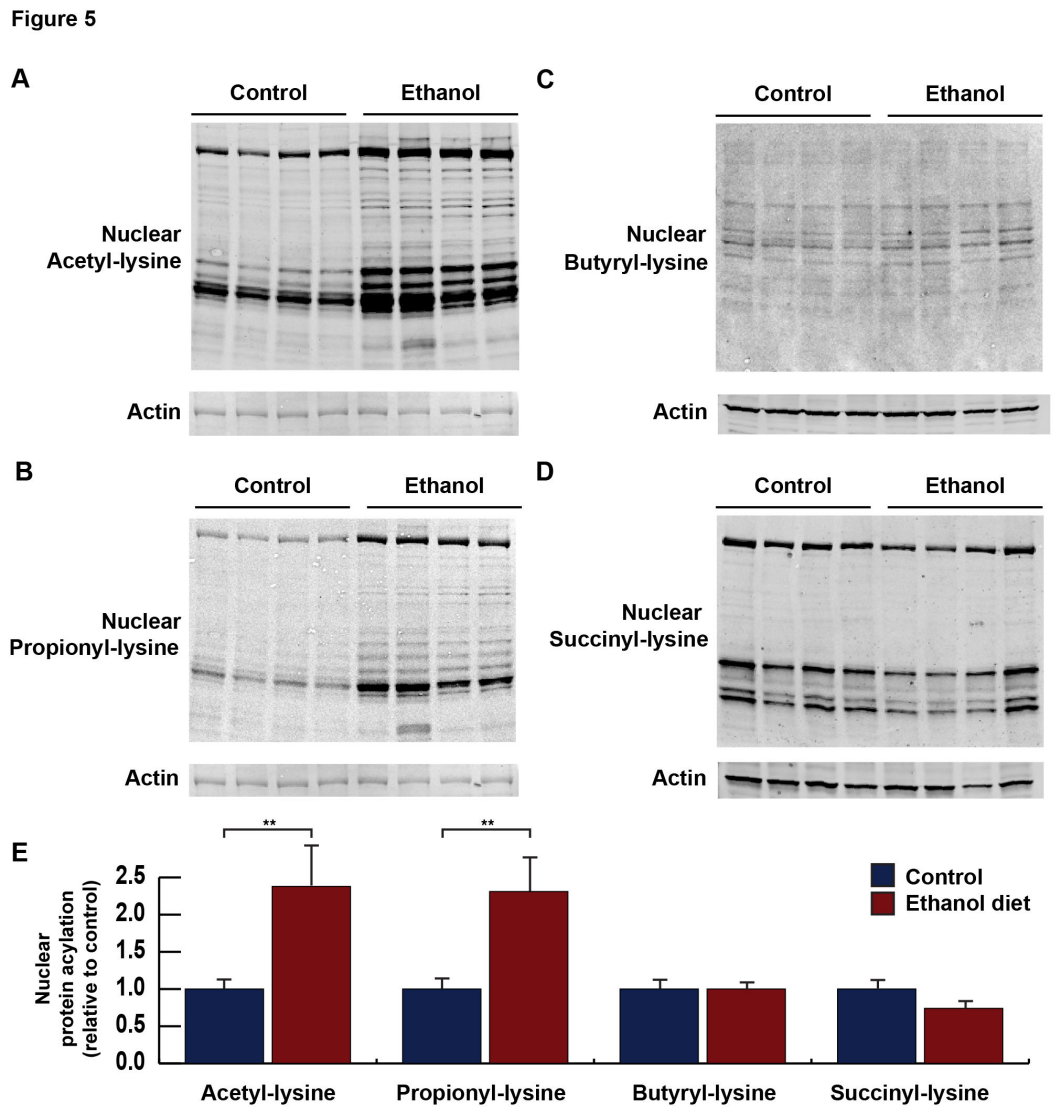
Nuclear extracts were isolated from livers of wild-type mice fed a standard or ethanol diet for 6-8 weeks and analyzed for total protein acetylation (A), propionylation (B), butyrylation (C), and succinylation (D) by western blot analysis with an acyllysine-specific antibody; normalized to total nuclear content using anti-lamin A/C; n = 4 mice/condition.

In addition to mitochondrial protein acetylation, nuclear protein acetylation has been shown to increase in response to ethanol diet feeding in mice [19]. Thus, we addressed the possibility that other acyl modifications were altered in the nucleus in response to ethanol diet feeding. Remarkably, similar changes in acyl modifications were observed in the nucleus as in the mitochondria. Specifically, nuclear protein acetylation and propionylation was elevated in response to ethanol ingestion (Figure 5A and 5B), compared to control diet feeding. When measuring protein butyrylation, the signal appeared higher in the nucleus compared to the mitochondria, but no changes were observed between standard diet and mice ingesting ethanol (Figure 5C). Finally, no large changes were observed in nuclear protein succinylation between standard diet and ethanol-containing diet (Figure 5D).

To gain a complete understanding of the sub-cellular protein acylation status during standard diet and chronic ethanol consumption, we also measured protein acetylation, propionylation, butyrylation, and succinylation in the cytoplasm (Figure S1) and the microsomes (Figure S2). In contrast to mitochondrial and nuclear compartments, protein acylation was generally unchanged between standard diet and ethanol diet feeding regimes in these sub-cellular compartments, and the overall signal was significantly lower.

### Identification of acylated proteins

**Figure 6:**
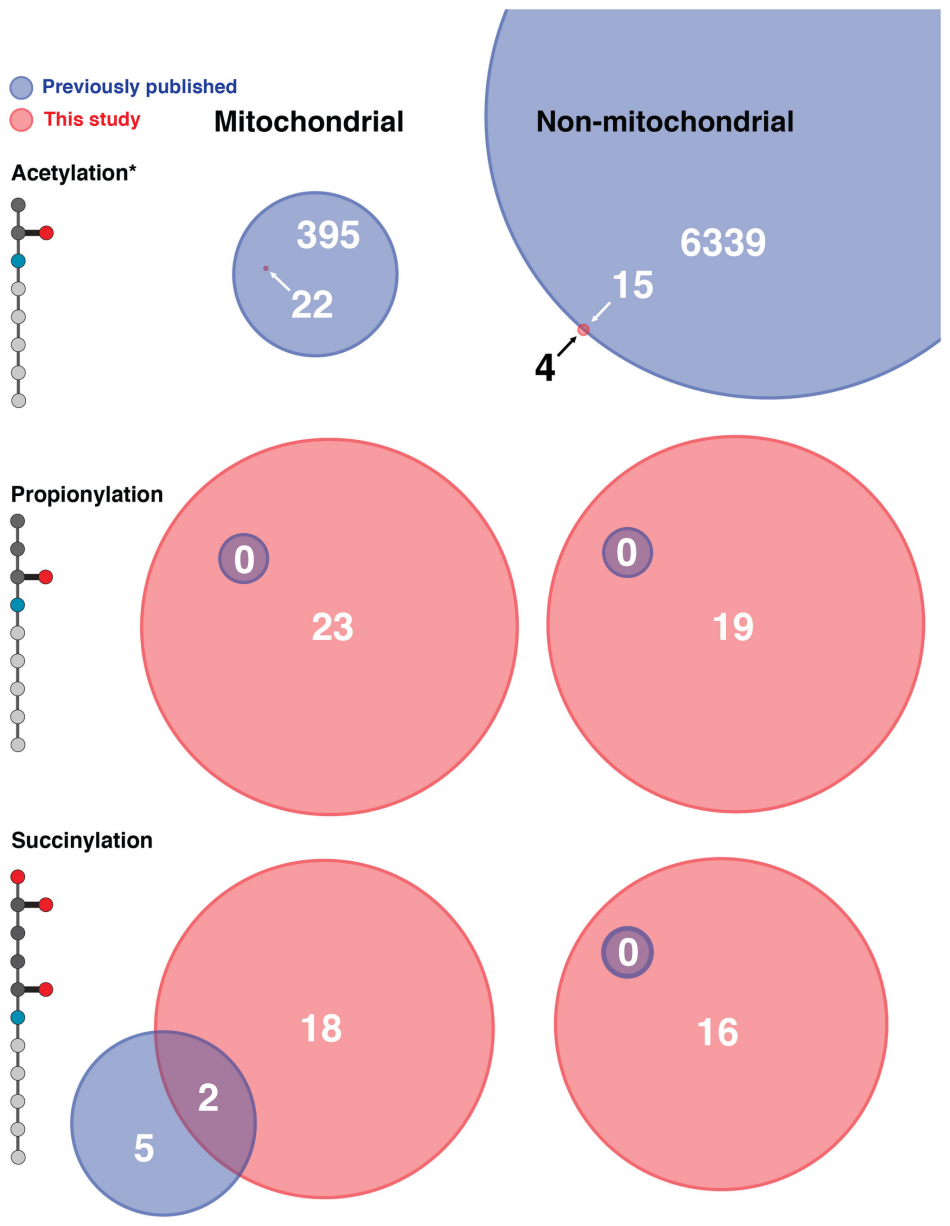
Proteomic data summary of all known acylated proteins identified in mitochondrial and non-mitochondrial compartments. Area represents number of proteins identified in this study (red), compared to previous studies (blue), as acetylated (10:1 scaling), propionylated, and succinylated.

To further probe the physiological role of these new acyl modifications, we took a proteomics approach to identify proteins containing the most abundant acyl modifications – namely acetylation, propionylation, and succinylation, in mitochondrial and nuclear compartments. We purified mitochondria and nuclear extracts from mice fed a standard diet or ethanol-containing diet, and subjected the lysates to immunoprecipitation using antibodies against acetyl-lysine, propionyl-lysine, and succinyl-lysine, as described above. The immunoprecipitated cell lysates were then resolved using 1D-SDS PAGE by running the protein a few centimeters into the resolving gel. Each protein lane was excised and trypsin digested followed by LC-MS/MS analysis. We found 22 acetylated proteins in the mitochondria, and 19 acetylated proteins in the non-mitochondrial (cytoplasmic and nuclear) compartments (Figure 6, Table S1). All of the acetylated proteins we found in the mitochondrial compartment have been previously identified in other proteomic acetylation surveys [29], and serves as a positive control for the use of this method to identify acylated proteins. However in non-mitochondrial compartments, we found four proteins immunoprecipitated with the acetyl-lysine antibody (*Pipox, Rpl14, Ugt1a1, Ugt2b17*), which have not been previously identified as acetylated. When analyzing immunoprecipitated proteins from the propionyl-lysine fraction, we found 23 proteins in the mitochondria, and 19 proteins in the non-mitochondrial compartments (Figure 6). When comparing these proteins to previously known propionylated proteins, none of the mitochondrial and non-mitochondrial proteins are known to be propionylated, demonstrating propionylated proteins are present in mammalian tissues. Finally, when analyzing immunoprecipitated proteins from the succinyl-lysine antibody, we found 20 proteins in the mitochondria, and 16 proteins in the non-mitochondrial compartments (Figure 6). This method found two proteins that had been previously reported to be succinylated in the mitochondria (*Cps1* and *Hmgcs2*), whereas 18 new proteins were identified. Together, these results reveal an increase in the number of known modifications on hepatic mitochondrial and non-mitochondrial proteins, and demonstrate these new acyl modifications are abundant in mammalian systems. Furthermore, mitochondrial enrichments of acetylated and propionylated peptides (Table 1) reveal the competing nature of lysine acylation and demonstrates that these modifications can occur on the same lysine residue of mitochondrial proteins.

**Table 1:** Site-specific identification of competing acetyl and propionyl lysine modifications in liver mitochondria from SIRT3 KO ethanol-fed mice.

**Accession**	**m/zmeas.**	**Mrcalc.**	**Δm/z[ppm**]	**Score**	**Sequence**	**Modification**	**AARange**
DHE3_MOUSE	508.35	1014.57	112.61	38	K.LVEDL**K**TR.E	Acetyl: 6	85-92
DHE3_MOUSE	515.37	1028.59	134.70	36.2	K.LVEDL**K**TR.E	Propionyl: 6	85-92
DHE3_MOUSE	718.90	1435.75	25.12	108.4	R.TAM**K**YNLGLDLR.T	Acetyl: 4	524-535
DHE3_MOUSE	725.94	1449.77	69.19	47.7	R.TAM**K**YNLGLDLR.T	Propionyl: 4	524-535
HMCS2_MOUSE	948.55	1894.95	69.43	84.4	K.FNNVEAG**K**YTVGLGQTR.M	Acetyl: 8	76-92
HMCS2_MOUSE	637.38	1908.97	77.82	36.3	K.FNNVEAG**K**YTVGLGQTR.M	Propionyl: 8	76-92
ATPA_MOUSE	580.87	1159.61	100.69	88.3	K.ISEQSDA**K**LK.E	Acetyl: 8	532-541
ATPA_MOUSE	587.87	1173.62	86.18	61.8	K.ISEQSDA**K**LK.E	Propionyl: 8	532-541
ALDH2_MOUSE	610.37	1218.66	52.93	67	K.F**K**TIEEVVGR.A	Acetyl: 2	429-438
ALDH2_MOUSE	617.33	1232.68	-25.14	30.2	K.F**K**TIEEVVGR.A	Propionyl: 2	429-438
THIL_MOUSE	851.47	1700.93	-5.54	96	R.GATPYGGV**K**LEDLIVK.D	Acetyl: 9	163-178
THIL_MOUSE	858.40	1714.95	-96.16	22.9	R.GATPYGGV**K**LEDLIVK.D	Propionyl: 9	163-178
AL4A1_MOUSE	572.33	1142.54	89.47	66.9	K.VA**K**F**C**YADK.A	Acetyl: 3	90-98
AL4A1_MOUSE	579.32	1156.56	57.62	22.8	K.VA**K**F**C**YADK.A	Propionyl: 3	90-98

### Pathway analysis of acylated proteins

**Figure 7:**
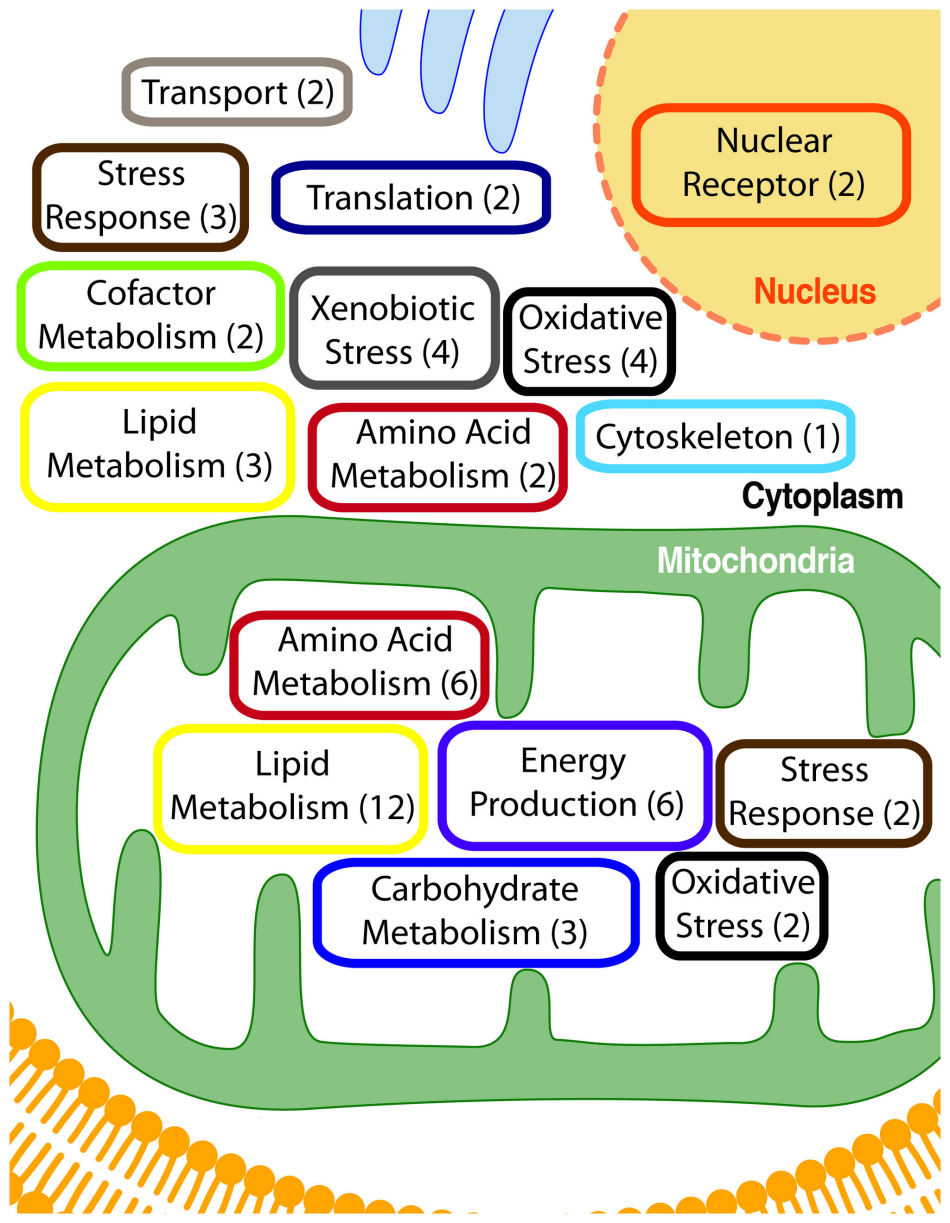
Pathway analyses of acylated proteins. Unique pathway (in box) and number of acylated proteins identified in that pathway (in parenthesis), divided by major sub-cellular compartments.

Remarkably, several of the same proteins were found with all three modifications, suggesting some pathways might use protein acylation as a common regulatory mechanism. To gain insight into the pathways that could be regulated by protein acylation, we performed a functional pathway analysis using the Database for Annotation, Visualization and Integrated Discovery (DAVID, v6.7) [30] of all proteins we identified as acetylated, propionylated, and succinylated by immunoprecipitating with modification-specific antibodies. We found proteins in several pathways were acylated, with an overrepresentation of metabolic processes (Figure 7). In particular, in the mitochondria we found amino acid metabolism (6 proteins), carbohydrate metabolism (3 proteins), energy production (6 proteins), lipid metabolism (12 proteins), oxidative stress response (2 proteins), and stress response (2 proteins) all contained acylated proteins. In non-mitochondrial compartments, these pathways were also identified as having acylated proteins, in addition to cofactor metabolism (2 proteins), cytoskeleton (1 protein), nuclear receptor (2 proteins), translation (2 proteins), transport (2 proteins), xenobiotic stress response (2 proteins). Together, a theme emerges for several metabolic and stress response pathways to be regulated by acetylation, and novel acyl modifications during ethanol metabolism.

## Discussion

Previous studies have shown protein acetylation changes in response to chronic ethanol ingestion [19,20]. Here, we have shown that in addition to acetylation, some, but not all, newly identified protein acyl modifications change during ethanol-diet feeding. Furthermore, these changes are regulated differently in mitochondrial, nuclear, cytoplasmic and microsomal compartments.

Notably, in the mitochondria we observed increases in protein acetylation and propionylation. SIRT3 is the primary deacetylase in the mitochondria, and may display reduced enzymatic activity upon ethanol consumption due modification of a zinc-binding cysteine by 4-hydroxynonenal (4-HNE) [6]. Because SIRT3 can remove propionyl-lysine *in vitro* [27], we tested the possibility that ablation of SIRT3 could explain the increase in propionylation. While some proteins in SIRT3KO mice showed increased propionylation by immunoblotting compared to wild-type mice during control diet feeding, the observed increases were not significantly different. These data suggest that the elevation in propionyl-lysine during ethanol feeding was not due to reduced SIRT3 activity, and more work is required to understand the genesis of the elevated modification. While the *in vivo* role for SIRT3 in regulating propionylation has not been described, the elevation of some bands in the SIRT3KO mice demonstrates SIRT3 could regulate this novel modification *in vivo*. Finally, in addition to reduced SIRT3 activity by 4-HNE, alterations in NAD^+^ levels could reduce SIRT3 activity and contribute to the increases in protein acetylation and propionylation [6].

In contrast to elevations in protein acetylation and propionylation, mitochondrial protein succinylation was reduced in response to ethanol-diet feeding. A reduction in the succinylation signal could suggest an upregulation of SIRT5, causing removal of this modification. However, a previous study in rats showed chronic ingestion of an ethanol-containing diet reduces SIRT5 levels [28]. In mice, we found no changes in SIRT5 protein levels; therefore, the reduction in succinylation is likely caused by other mechanisms and further work is required to explain this observation.

The trends in changes in acylation of proteins in the nucleus were similar to those observed in the mitochondria following ethanol consumption. Specifically, protein acetylation and propionylation were elevated in the nucleus, while butyrylation was unchanged, and succinylation was modestly reduced (Figure 5). While acetylation, and its regulation by histone deacetylases (HDAC1-11) and the nuclear sirtuins (SIRT1, 6, and 7), has been well studied in the nucleus (for a review, see 31), nothing is yet known about these novel acyl modifications. One recent study reported histone proteins are malonylated and succinylated [32]. Thus, the data presented here shows propionyl-, butyryl- and succinyl-modifications are present on non-histone proteins; however, more work is required to understand their physiological roles.

In contrast to the mitochondrial and nuclear compartments, no significant changes were observed in the acyl protein modifications in the cytoplasm and microsomal compartments, suggesting that these modifications are not relevant during ethanol diet feeding, in wild-type or SIRT3KO mice (Figures S3-S5). However, the presence of these modifications alone suggests that they could be relevant under different metabolic conditions.

A major outstanding question in the protein acylation field surrounds the mechanism of acylation. Acetyl-CoA levels could be elevated during ethanol metabolism due to production from the enzymatic oxidation of ethanol [7], and thus this increase could drive the increase in protein acetylation. Alternatively, the recently identified component of the mitochondrial acetyltransferases machinery (MAT1), could also be playing a role [33]. However, propionyl-CoA is not generated directly from ethanol metabolism, but instead is produced from amino acid metabolism and odd-chain fatty acid metabolism. Thus, the mechanism leading to the increase in protein propionylation could be occurring through the activation of these pathways or through an unidentified path from ethanol to propionyl-CoA. The reduction in mitochondrial protein succinylation could be explained through three possible mechanisms. First, ethanol metabolism is known to alter the NAD^+^/NADH ratio, and suppress the TCA cycle, resulting in reduction of the succinyl-CoA pool [7]. Second, if acetylation and succinylation are occurring on the same lysine residues, then the marked increase in acetylation could out-compete the succinyl modification for the same sites. Third, the reduction in succinylation could be a direct regulatory mechanism to help the cell cope with the ethanol-induced stress. One caveat to understanding the complex nature of these acyl modifications is a heavy reliance on antibody enrichment. Antibody specificity and reproducibility vary widely among developers and epitope target (i.e. acetyl, propionyl, and succinyl), further complicating comparisons of different acyl modifications. Current efforts are underway to fully understand the mechanism leading to changes in novel acyl modifications, independent of the sirtuin deacylases.

In summary, we have shown that novel acyl post-translational modifications are present in hepatic mitochondrial, nuclear, cytoplasmic, and microsomal compartments and ethanol ingestion induces specific changes in these acyl modifications. Surprisingly, these changes are independent of sirtuin expression. Future directions will be aimed at identifying the mechanisms leading to acylation, as well as identifying the specific sites of acylation by mapping the acyl landscape with higher resolution. In conclusion, these early studies suggest that protein acylation, beyond protein acetylation, contributes to the overall metabolic regulatory network and is an emerging regulatory mechanism during sustained ethanol consumption and metabolism.

## Materials and Methods

### Animal Models

All procedures involving animals were approved by the Institutional Animal Care and Use Committee of the University of Colorado and were performed in accordance with published National Institutes of Health guidelines. Male, wild-type (WT) or *Sirt3* ^*-/-*^ (KO) C57Bl/6J mice were obtained from Dr. Eric Verdin (Gladstone Institutes, San Francisco, CA). For ethanol feeding studies, mice were fed a modified Lieber-DeCarli liquid based-diet (Bio-Serv, Frenchtown, NJ) for 6 weeks. The diet consisted of 44% fat-derived calories, 16% protein-derived calories and the remaining balance being comprised of either carbohydrate or ethanol-derived calories. Ethanol-fed mice began the study on a diet consisting of 2% (v/v) ethanol, with the ethanol-derived calories increased on a weekly basis until sacrifice; week 6 consisted of 6% ethanol (v/v) or 31.8% ethanol-derived calories. Pair-fed animals were each matched to an ethanol-fed mouse where ethanol-derived calories were replaced with calories from a carbohydrate source (maltose-dextrin). Upon completion of the study, animals were anesthetized via intraperitoneal injection of sodium pentobarbital and euthanized via exsanguination. Livers were excised, weighed, and subjected to differential centrifugation using a sucrose gradient for subcellular fractionation to obtain cytosolic, microsomal and mitochondrial enriched fractions, as previously described [34]. Nuclear fractions were isolated using a well-characterized kit (Active Motif, Carlsbad, CA).

### Immunoblotting for acylated proteins

Protein from sub-cellular fractionations were separated by standard SDS-PAGE and transferred to a Hybond-P membrane (GE Healthcare, Buckinghamshire, UK). Membranes were blocked for 30-60 minutes with TBST and 5% NFDM or 5% milk in PBS/0.1% Tween. Membranes were then probed with primary antibodies directed against acetyllysine (Cell Signaling, Boston, MA or PTM Biolabs, Inc., Chicago, IL), propionyllysine (PTM Biolabs), butyryllysine (PTM Biolabs), succinyllysine (PTM Biolabs), ETF (Gift from Eric Goetzman and Jerry Vockley, Children's Hospital of Pittsburgh), VDAC (Abcam), β-actin (Sigma), PDI (Cell Signaling Technology, Danvers, MA), Lamin B1 (Abcam), SIRT3 (Gift from Eric Verdin), SIRT4 (Abcam) and SIRT5 (Abcam). Following 3 washes with TBST or PBS/0.1% Tween, a horseradish peroxidase or near-infrared conjugated secondary was applied and membranes were imaged according to standard procedures. Band intensities were quantified using Image Studio (Li-cor, Omaha, NE) or ImageJ and normalized to the protein loading control.

### Immunoprecipitation of Acylated Proteins

Mitochondrial protein (500 µg) was incubated with antibodies directed against acetylated-lysine epitopes (5.0 µL, PTM Biolabs) overnight at 4 °C. 100 µL of protein G agarose (Sigma) was then applied to the samples and allowed to rotate overnight at 4 °C. Supernatants were removed and the beads were washed 5 times with TBST. Samples were boiled for 5 minutes in SDS loading buffer and then applied to 10% SDS-PAGE gels, where the sample were run into the gel approximately 3 cm. The gels were then coomassie stained for 10 min and destained overnight. Protein bands were excised from the gel and digested with trypsin, as previously described [6]. Additionally, acetyl and propionyl peptide enrichment was utilized following the manufacturers protocol (PTM Biolabs).

### LC-MS/MS Identification of Mitochondrial Acetylated Proteins

Protein digests were analyzed using nanoliquid chromatography (EASY-nLC, Proxeon) at a flow rate of 300 nL/min with a gradient of 5 to 40% ACN (0.2% formic acid) over 40 min on C-18 Proxeon EASY-Column trapping (20 x 0.1 mm) and analytical columns (100 x 0.075 mm). The nLC was coupled to a nano-ESI source and Esquire HCT ion trap mass spectrometer (Bruker Daltonics, Inc., Billerica, MA). The instrument was operated using data-dependent CID MS/MS with a threshold of 30,000 TIC. Data analysis was performed using Mascot (version 2.2.04, www.matrixscience.com). Mascot generic files were then imported into Scaffold (version 3.3.3, Proteome Software, Inc., Portland, OR) to analyze, interpret and organize the MS/MS data. Scaffold analysis included only protein IDs with a probability of 99% or greater and peptide IDs required a 95% cutoff. Searches resulted in the assignment of a UniProt (*Universal Protein Resource* http://www.uniprot.org/) accession number which was used for subsequent analysis.

### Statistical Analyses

Statistical analysis and generation of graphs was performed using GraphPad Prism 4.02 (GraphPad Software, San Diego, CA). Differences between the four treatment groups were assessed using a one-way ANOVA analysis with a Tukey post-test; statistical significance was determined if *p* < 0.05.

## Supporting Information

Figure S1 **Cytoplasmic extracts were isolated from livers of wild-type mice fed a standard or ethanol diet for 6-8 weeks and analyzed for total protein acetylation (A), propionylation (B), butyrylation (C), and succinylation (D) by western blot analysis with an acyl-lysine-specific antibody; normalized to total cytoplasmic content using anti-beta-actin; n = 4 mice/condition.**
(TIF)

Figure S2 **Microsomal extracts were isolated from livers of wild-type mice fed a standard or ethanol diet for 6-8 weeks and analyzed for total protein acetylation (A), propionylation (B), butyrylation (C), and succinylation (D) by western blot analysis with an acyl-lysine-specific antibody; normalized to total microsomal content using anti-protein disulfide isomerase (PDI); n = 4 mice/condition.**
(TIF)

Figure S3 **Nuclear extracts were isolated from livers of wild-type or SIRT3KO mice fed a standard or ethanol diet for 6-8 weeks and analyzed for total protein acetylation (A), propionylation (B), butyrylation (C), and succinylation (D) by western blot analysis with an acyllysine-specific antibody; normalized to total nuclear content using anti-Lamin B; n = 4 mice/condition.**
(TIFF)

Figure S4 **Cytoplasmic extracts were isolated from livers of wild-type or SIRT3KO mice fed a standard or ethanol diet for 6-8 weeks and analyzed for total protein acetylation (A), propionylation (B), butyrylation (C), and succinylation (D) by western blot analysis with an acyllysine-specific antibody; normalized to total cytoplasmic content using anti-actin; n = 4 mice/condition.**
(TIF)

Figure S5 **Microsomal extracts were isolated from livers of wild-type or SIRT3KO mice fed a standard or ethanol diet for 6-8 weeks and analyzed for total protein acetylation (A), propionylation (B), butyrylation (C), and succinylation (D) by western blot analysis with an acyllysine-specific antibody; normalized to total microsomal content using anti-protein disulfide isomerase (PDI); n = 4 mice/condition.**
(TIF)

Table S1 **LC-MS/MS analyses of acylated peptides of mitochondrial proteins: immunoprecipitated using acyl-lysine antibodies, 1D-SDS-PAGE, band excision and trypsin digestion followed by LC-MS/MS analysis.**
(XLSX)

Table S2 **Complete protein and peptide list for the site-specific identification of competing acetyl and propionyl lysine modifications in liver mitochondria from SIRT3 KO ethanol-fed mice.**
(XLS)
