# Supplementary figures and images for: Ethanol Metabolism Modifies Hepatic Protein Acylation in Mice
Source: PLoS One. 2013 Sep 20;8(9):e75868. doi: 10.1371/journal.pone.0075868 (PMC3779192; doi:10.1371/journal.pone.0075868)

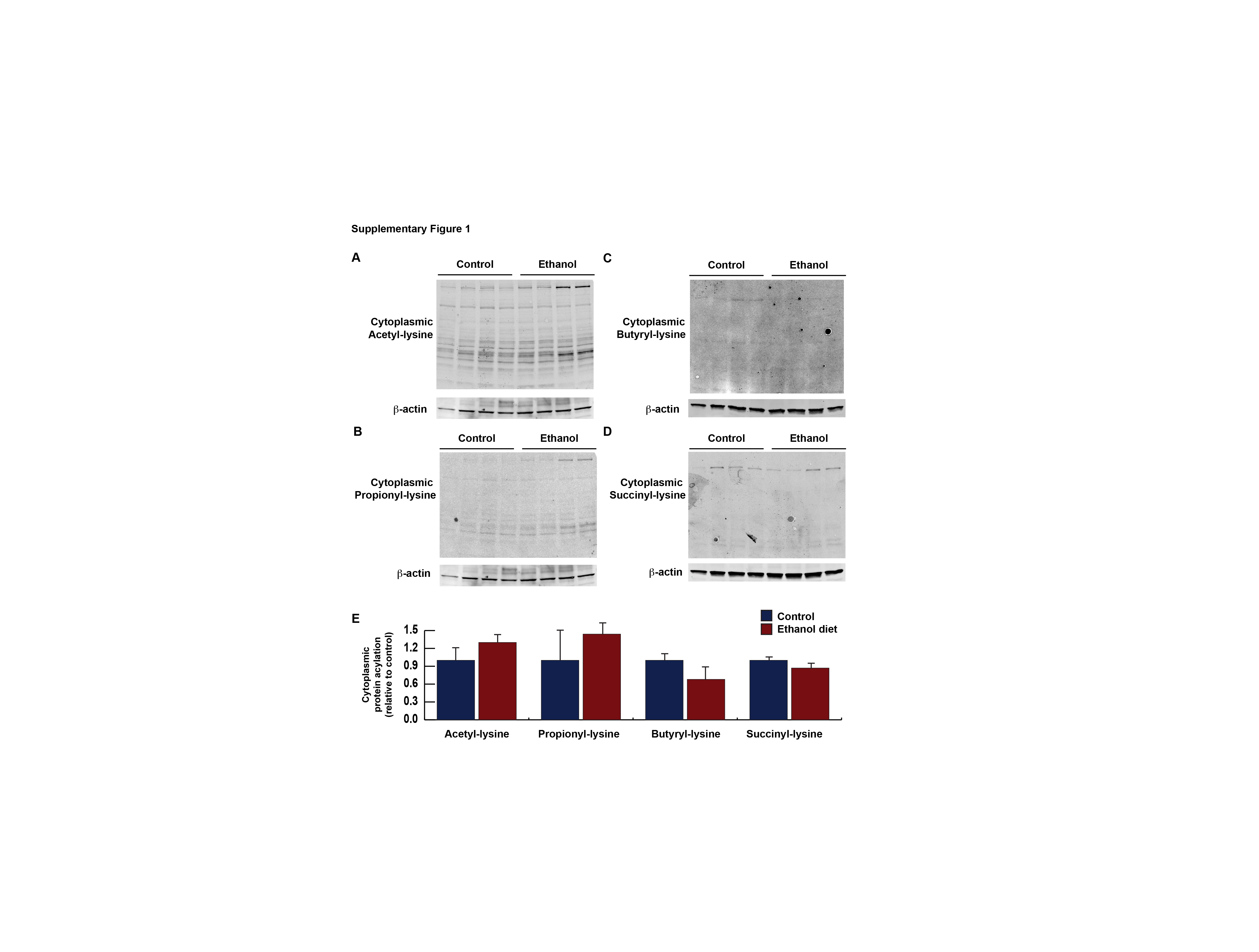

Supplement: Figure S1 — Cytoplasmic extracts were isolated from livers of wild-type mice fed a standard or ethanol diet for 6-8 weeks and analyzed for total protein acetylation (A), propionylation (B), butyrylation (C), and succinylation (D) by western blot analysis with an acyl-lysine-specific antibody; normalized to total cytoplasmic content using anti-beta-actin; n = 4 mice/condition. (TIF) [file pone.0075868.s001.tif]

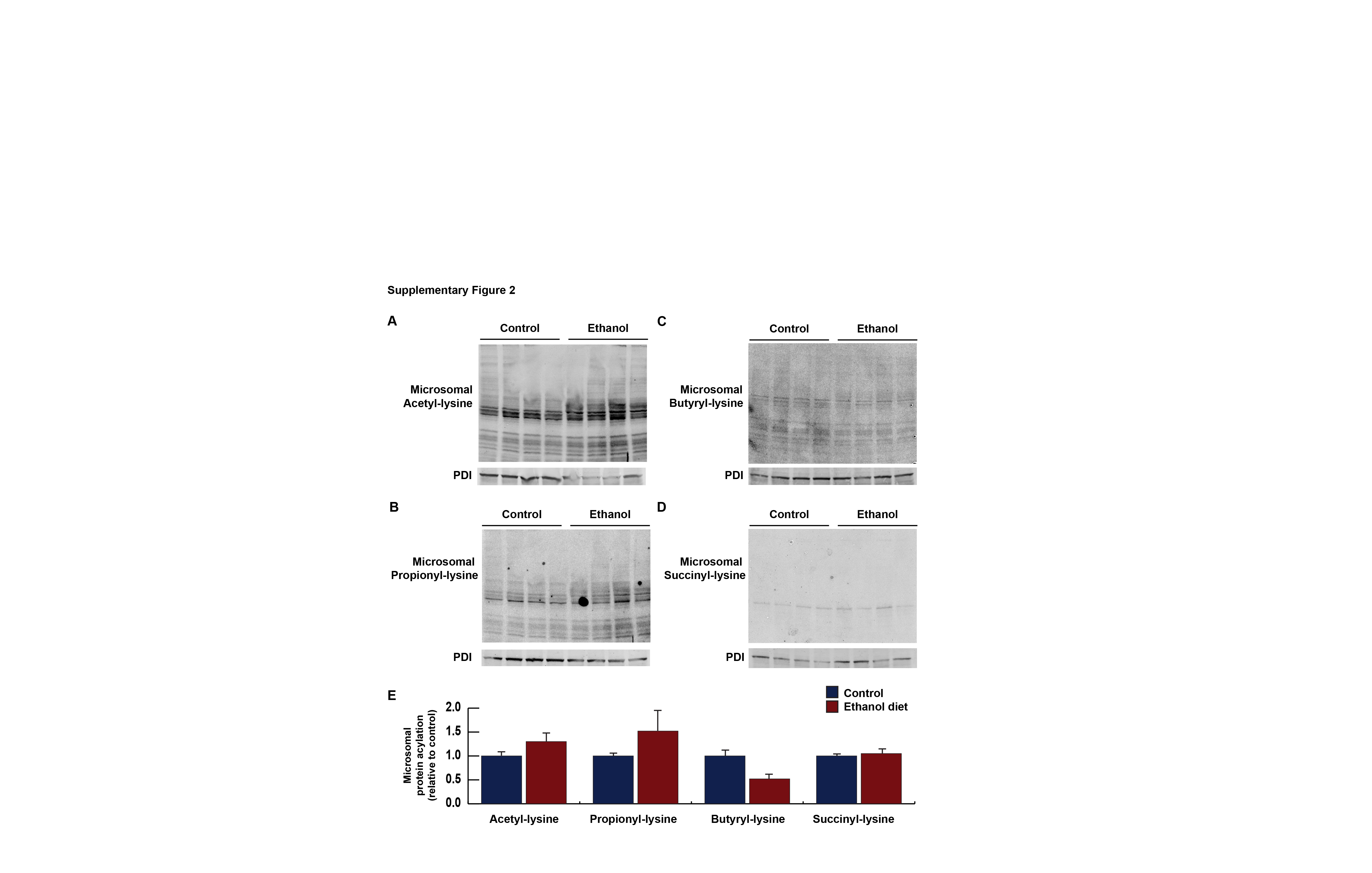

Supplement: Figure S2 — Microsomal extracts were isolated from livers of wild-type mice fed a standard or ethanol diet for 6-8 weeks and analyzed for total protein acetylation (A), propionylation (B), butyrylation (C), and succinylation (D) by western blot analysis with an acyl-lysine-specific antibody; normalized to total microsomal content using anti-protein disulfide isomerase (PDI); n = 4 mice/condition. (TIF) [file pone.0075868.s002.tif]

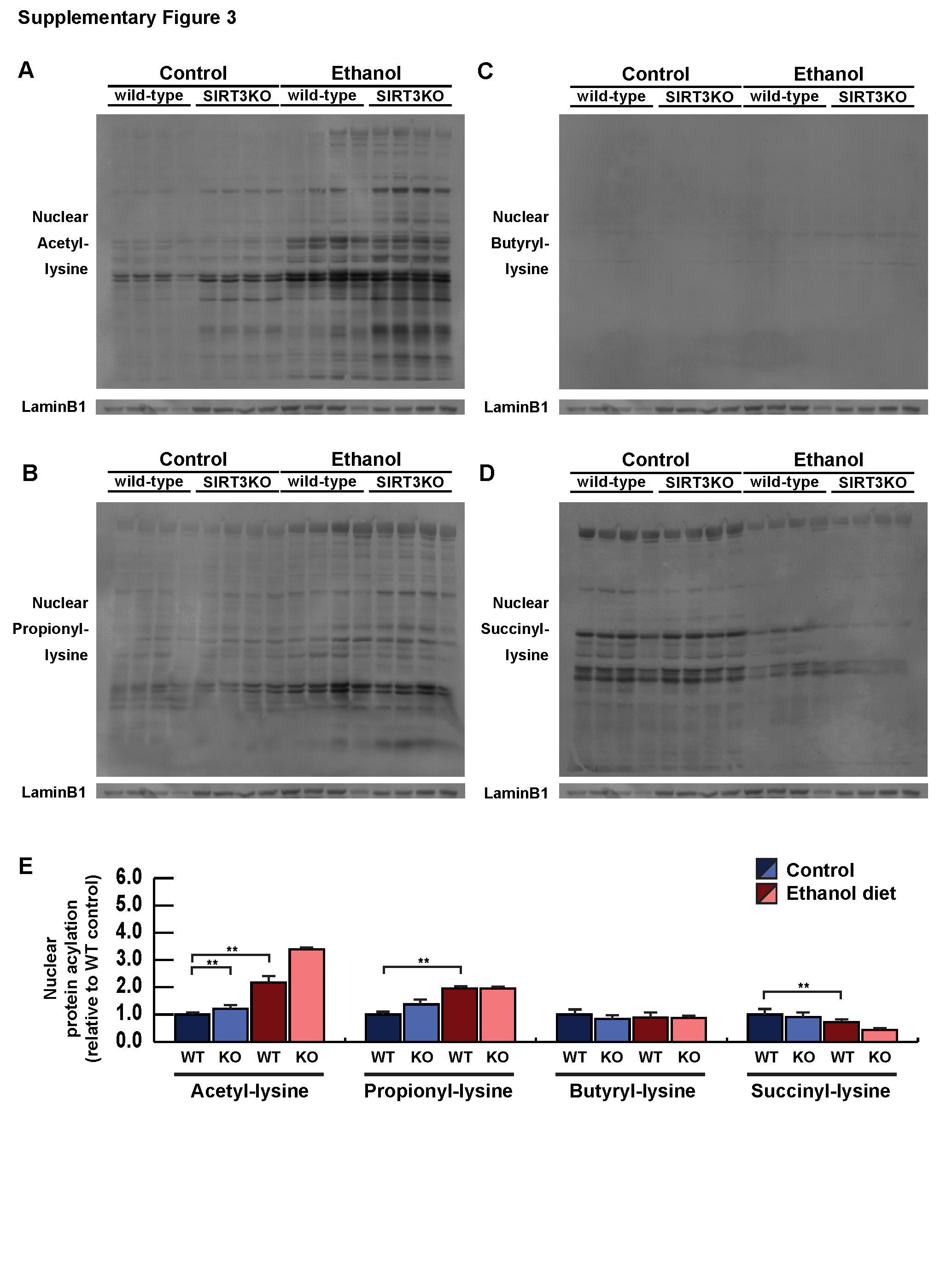

Supplement: Figure S3 — Nuclear extracts were isolated from livers of wild-type or SIRT3KO mice fed a standard or ethanol diet for 6-8 weeks and analyzed for total protein acetylation (A), propionylation (B), butyrylation (C), and succinylation (D) by western blot analysis with an acyllysine-specific antibody; normalized to total nuclear content using anti-Lamin B; n = 4 mice/condition. (TIFF) [file pone.0075868.s003.tiff]

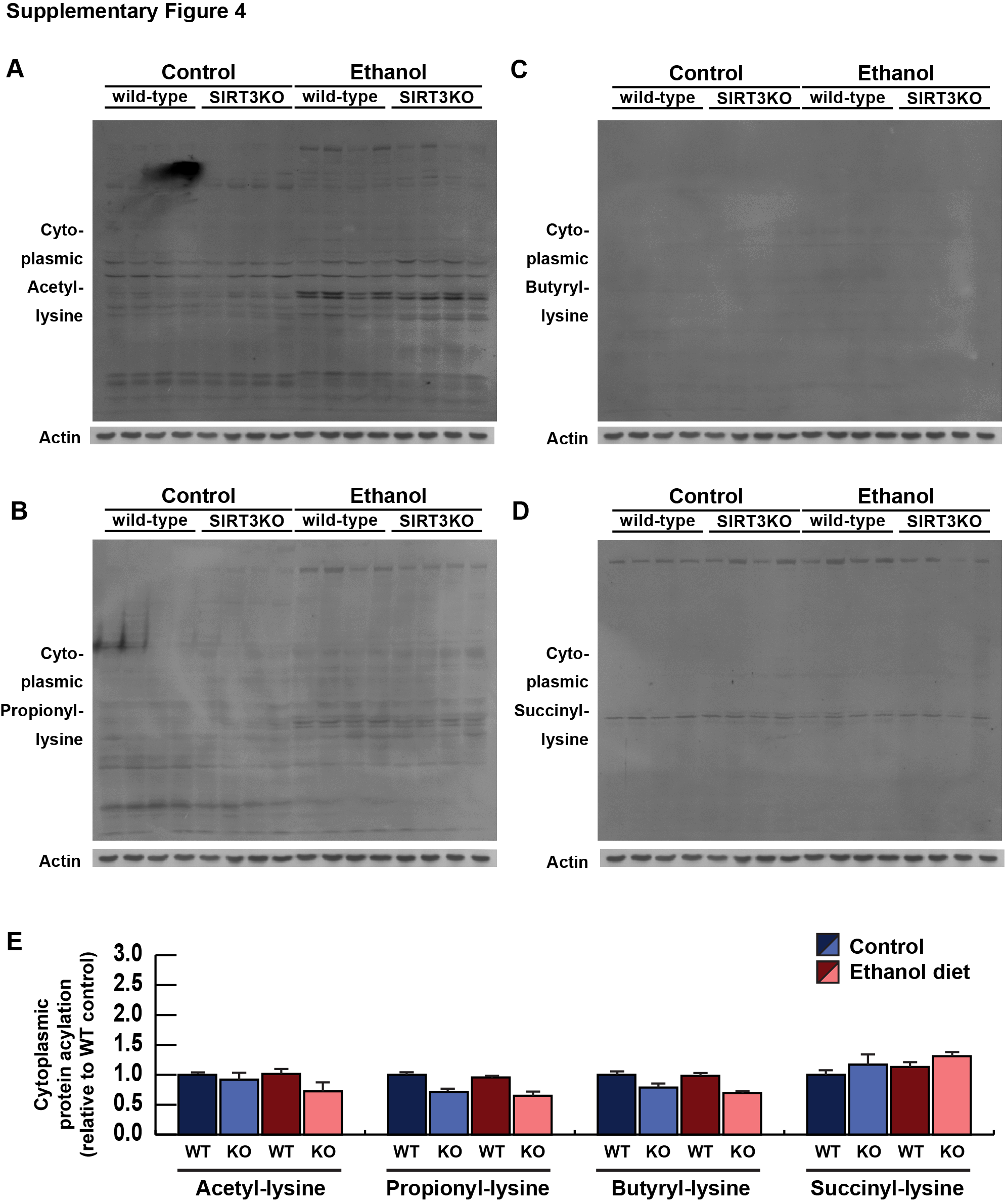

Supplement: Figure S4 — Cytoplasmic extracts were isolated from livers of wild-type or SIRT3KO mice fed a standard or ethanol diet for 6-8 weeks and analyzed for total protein acetylation (A), propionylation (B), butyrylation (C), and succinylation (D) by western blot analysis with an acyllysine-specific antibody; normalized to total cytoplasmic content using anti-actin; n = 4 mice/condition. (TIF) [file pone.0075868.s004.tif]

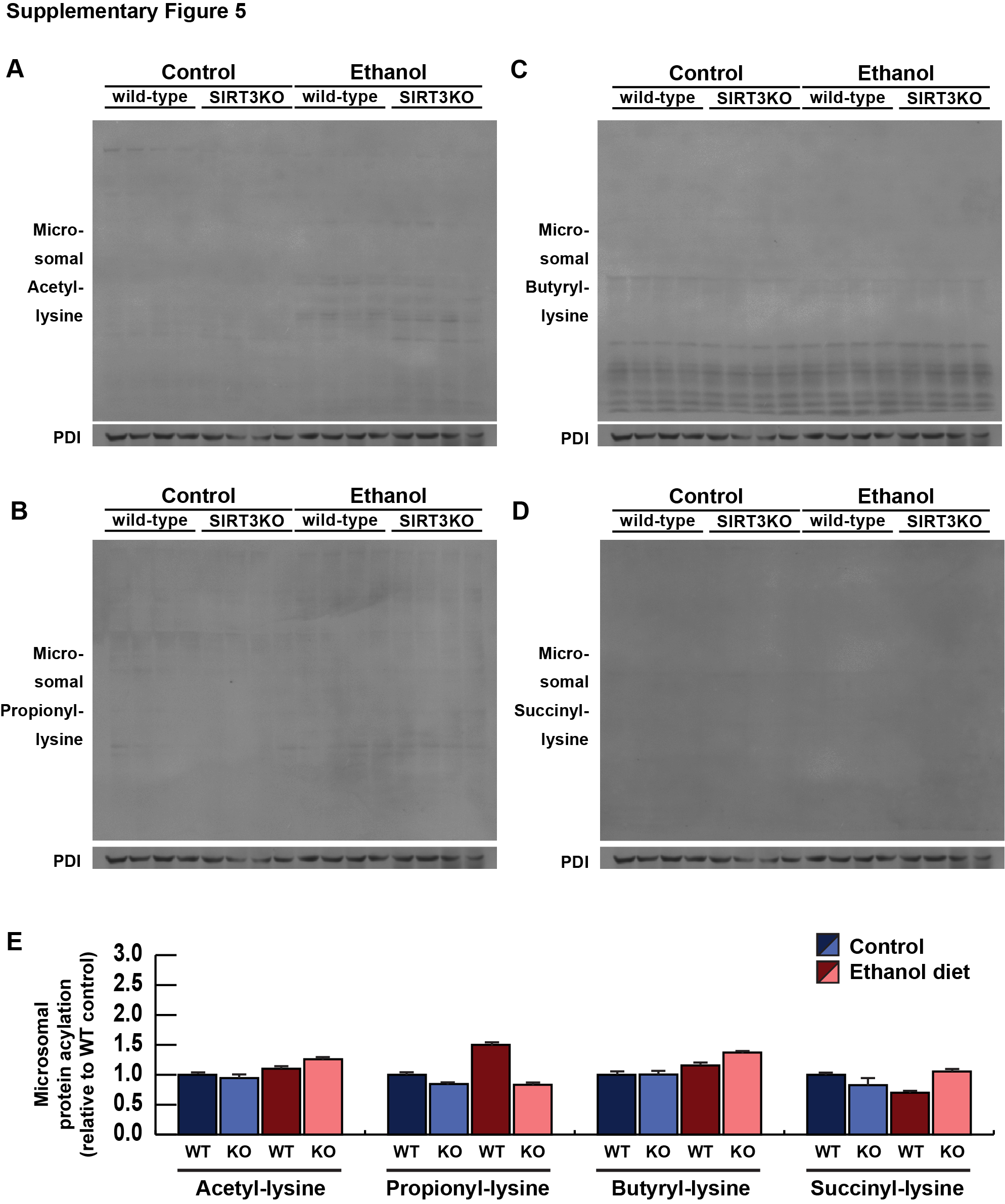

Supplement: Figure S5 — Microsomal extracts were isolated from livers of wild-type or SIRT3KO mice fed a standard or ethanol diet for 6-8 weeks and analyzed for total protein acetylation (A), propionylation (B), butyrylation (C), and succinylation (D) by western blot analysis with an acyllysine-specific antibody; normalized to total microsomal content using anti-protein disulfide isomerase (PDI); n = 4 mice/condition. (TIF) [file pone.0075868.s005.tif]
